# Clinical Evaluation of a Metagenomics-Based Assay for Pneumonia Management

**DOI:** 10.3389/fmicb.2021.751073

**Published:** 2021-09-16

**Authors:** Yangqing Zhan, Teng Xu, Fusheng He, Wei-jie Guan, Zhengtu Li, Shaoqiang Li, Mingzhou Xie, Xiaolei Li, Rongchang Chen, Linling Cheng, Nanshan Zhong, Feng Ye

**Affiliations:** ^1^State Key Laboratory of Respiratory Disease, Department of Pulmonary and Critical Care Medicine, The First Affiliated Hospital of Guangzhou Medical University, Guangzhou Institute of Respiratory Health, National Clinical Research Center for Respiratory Disease, Guangzhou, China; ^2^Vision Medicals Co., Ltd., Guangzhou, China; ^3^Key Laboratory of Animal Gene Editing and Animal Cloning in Yunnan Province and College of Veterinary Medicine, Yunnan Agricultural University, Kunming, China; ^4^Department of Thoracic Surgery, Guangzhou Institute for Respiratory Health, The First Affiliated Hospital of Guangzhou Medical University, Guangzhou, China; ^5^Department of Respiratory and Critical Care Medicine, First Affiliated Hospital of Southern University of Science and Technology, Second Clinical Medical College of Jinan University, Shenzhen People’s Hospital, Shenzhen Institute of Respiratory Diseases, Shenzhen, China

**Keywords:** pneumonia, metagenomic next-generation sequence, pathogens, cytomegalovirus, *pneumocystis jirovecii*

## Abstract

Clinical value of metagenomic next-generation sequencing (mNGS) in pneumonia management is still controversial. A prospective study was conducted to evaluate the clinical impact of PneumoSeq in 57 immunocompetent (ICO) and 75 immunocompromised (ICH) pneumonia patients. The value of PneumoSeq for both etiological and clinical impact investigation in pneumonia was assessed. Among the 276 potential pathogens detected with PneumoSeq in our cohort, 251 (90.9%) were cross-validated. Clinical diagnoses of the causative pathogens were obtained for 97 patients, 90.7% of which were supported by PneumoSeq. Compared to conventional testing, PneumoSeq suggested potentially missed diagnoses in 16.7% of cases (22/132), involving 48 additional pathogenic microorganisms. In 58 (43.9%) cases, PneumoSeq data led to antimicrobial treatment de-escalation (*n* = 12 in ICO, *n* = 18 in ICH) and targeted treatment initiation (*n* = 7 in ICO, *n* = 21 in ICH). The PneumoSeq assay benefited the diagnosis and clinical management of both ICH and ICO pneumonia patients in real-world settings.

## Introduction

Pneumonia is among the most common infectious diseases leading to hospitalization (Prina et al., 2015; Ramirez et al., 2017; Torres et al., 2019) and contributes to significant mortality and morbidity (Barriere, 2010; Torres et al., 2019). According to the World Health Organization (WHO, 2020), pneumonia causes 3 million deaths each year worldwide. The timely and accurate detection of the causative pathogens is crucial for effective tailored clinical management. However, due to the atypical manifestations of pneumonia in immunocompromised hosts (ICHs), empirical management is often ineffective in guiding the appropriate treatment of pneumonia in these patients (Sousa et al., 2013; Azoulay et al., 2019; Di Pasquale et al., 2019). The high prevalence of opportunistic pathogens (e.g., cytomegalovirus (CMV), *Pneumocystis jirovecii*, fungi, *Nocardiosis*) and concurrent infections, the difficulty of effectively identifying pathogens based on clinical manifestations, and the limitations of conventional diagnostic techniques have further complicated the effective clinical diagnosis and treatment of pneumonia in ICH patients, leading to failures in the timely administration of targeted antimicrobial drugs. As a result, the mortality rate in ICH patients with pneumonia has remained higher than that in immunocompetent (ICO) patients (Letourneau et al., 2014). Hence, there is an urgent need to develop a diagnostic technique that can rapidly detect a wide range of pathogens in a single test and lead to personalized treatment, especially for immunocompromised host with pneumonia (Chiu and Miller, 2019; Di Pasquale et al., 2019; Azoulay et al., 2020).

Metagenomic next-generation sequencing (mNGS) has emerged as a promising, culture-independent approach for detecting potentially pathogenic microorganisms (Naccache et al., 2014; Simner et al., 2018; Dulanto Chiang and Dekker, 2020; Ramachandran and Wilson, 2020). Unlike assays that target specific genes of pathogens or certain ribosomal RNA amplicons, mNGS is unique in its ability to detect a wide array of bacteria, viruses, and fungi in an unbiased manner (Naccache et al., 2014; Goldberg et al., 2015; Forbes et al., 2017; Chiu and Miller, 2019; Gu et al., 2019). Data have shown that mNGS can improve the diagnosis of neurologic infections and provide actionable information in some cases (Wilson et al., 2019). However, the application of mNGS for respiratory pathogen detection is challenging due to the wide variety and high abundance of microbes within airways. Fungal infection is also common among ICH patients (Di Pasquale et al., 2019). In theory, mNGS, which take a short turnout time, and detect all microorganisms at one time, is an ideal detection method for patients with ICH pneumonia to determine the etiology as soon as possible and to carry out targeted individualized treatment. Nevertheless, there is currently a lack of prospective studies assessing the use of mNGS for guiding the etiology diagnosis and treatment of pneumonia in ICH patients (Zinter et al., 2019a).

We hypothesized that an optimized mNGS assay could improve the clinical management of pneumonia. Our study aimed to better understand the microbial etiology of pneumonia in ICO and ICH patients and the value of PneumoSeq in the clinical management of pneumonia (especially in ICH patients).

## Materials and Methods

### Patients and Samples

This prospective study was conducted in the Department of Pulmonary and Critical Care Medicine, the First Affiliated Hospital of Guangzhou Medical University, between April 2019 and January 2020. Consecutive patients older than 14 years with community acquired pneumonia (Cao et al., 2018) or hospital acquired pneumonia (Shi et al., 2019) were enrolled and divided into ICH or ICO group according to the immune status of the patients, with details shown in Table 1. This study was approved by the ethics committee of the First Affiliated Hospital of Guangzhou Medical University (No. 2019-49), and written informed consent was obtained from all subjects or their guardians.

**TABLE 1:** Definition of the study population.

**Items**	**Criteria**
Study population	Immunocompromised host (ICH): pneumonia patients who meed the criteria of immunocompromised status
	Immunocompetent host (ICO): pneumonia patients who do not meet the criteria of immunocompromised status.
Immunocompromised status	Presence of one of the following diseases and/or treatments:
	i. solid-organ or hematopoietic cell transplantation
	ii. neutropenia
	iii. solid or hematological malignancy under treatment
	iv. any type of known immunodeficiency disease
	v. receiving more than 0.3 mg/kg.d systemic corticosteroids for more than 3 weeks within 60 days or any type of immunosuppressive drugs (such as methotrexate, azathioprine, cyclosporine, or cyclophosphamide)
Inclusion criteria	Community acquired pneumonia, fulfill all clinical features as follow:
	i. community onset
	ii. a new infiltrate, lobar or segment consolidation, ground glass opacity or interstitial images is revealed on chest radiograph or CT scan, accompanied by pleural effusion or not
	iii. any of the following 4 clinical features: (I) new occurrence of cough, expectation, or worsen of respiratory symptoms, accompanied by purlurent sputum, chest pain, dyspnea and hemoptysis or not; (II) fever; (III) signs of consolidation and/or moist rale on lung auscultation; (IV) peripheral white cell counts > 10 × 10^9/L or < 4 × 10^9/L.
	Hospital acquired pneumonia, fulfill all clinical features as follow:
	i. a new or progressive infiltrate, consolidation, or ground glass opacity is revealed on chest radiograph or CT scan
	ii. two or more of the following 3 criteria: (I) fever > 38 °C; (II) purulent airway secretions; (III) peripheral white blood cell count > 10 × 10^9/L or < 4 × 10^9/L
	iii. illness occurs 48 hours or more after admission during hospitalization.
Exclusion criteria	i. patients infected with HIV
	ii. pregnant women
	iii. patients with an irreversible contraindication for bronchoscopy
	iv. patients who were unable to understand the informed consent description or unwilling to sign the informed consent form
	v. patients ultimately diagnosed with a non-infectious disease other than pneumonia

All patients received conventional diagnostic methods for identification of pathogens based on the patient’s condition during hospitalization. The choice of conventional diagnostic methods was determined by physician and conventional diagnostic methods could be repeated if necessary. The most common conventional diagnostic methods for microbiological diagnosis were culture for bacteria and fungi, polymerase chain reaction (PCR)-based assays for common respiratory viruses and mycoplasma pneumoniae, and serum tests for fungal infections. Specimen types included throat swabs, sputum, bronchoalveolar lavage fluid (BALF), blood, and pleural effusion, etc. Throat swab was only used for detection of respiratory viruses and atypical pathogens via PCR, including influenza virus type A and B, parainfluenza virus type 1/2/3, adenovirus, respiratory syncytial virus, mycoplasma pneumoniae, chlamydia pneumoniae and legionella pneumophila. Surplus of the sputum and BALF after conventional diagnostic methods was tested by PneumoSeq. In addition, throat swabs and blood were also collected and tested by PneumoSeq to match sample types of the conventional diagnostic methods. Another purpose of selection of throat swabs for PneumoSeq was to compare the role of different specimen types in pathogenic diagnosis. Additional PCR assays were retrospectively conducted for the validation of the positive PneumoSeq results if the index patient didn’t receive PCR testing during hospitalization.

### Sample Preparation, Library Construction and Sequencing for PneumoSeq

Whole-blood samples were collected in Geneseek serum cell-free DNA tubes and transported at ambient temperature. Other specimens were stored and transported at 4–8°C after collection. Prior to extraction, BALF and homogenized sputum specimens were centrifuged at 8,000 *g* for 5 min before being resuspended in lysis buffer with 0.1% sodium dodecyl sulfate (SDS) and 1% Non-idet P-40 (NP40). The dried oropharyngeal swab samples were soaked in 1 ml of sterile PBS for 10 min. Plasma was separated from whole blood by centrifugation at 500 *g* for 5 min. For DNA extraction, 600 μL of the processed specimens was mixed with glass beads of 0.1–0.2 mm diameter. A vortex mixer (Crystal, TX, United States) was used to disrupt the bacterial cell wall at 1,600 *g* for 10 min. The tubes were then heated at 99°C for 10 min before DNA extraction. RNA extraction was performed in parallel. The concentration of extracted DNA/RNA was measured using a Qubit Fluorometer before library preparation.

DNA libraries were prepared via transposase-based methodology. Human rRNA was depleted from the RNA samples via an RNase H-based method before library preparation. After purification and size selection, the concentration of the RNA library was determined by using a Qubit instrument before pooling. Pooled libraries were sequenced on an Illumina NextSeq 550 system using a 75 bp, single-end sequencing kit (Illumina, San Diego). The qualified results had no fewer than 15 million reads obtained per sample and a Q30 score of 90% or greater. A negative control sample was processed and sequenced in parallel in each sequencing run for quality control.

### Bioinformatic Pipeline for PneumoSeq

The 75bp single-end reads from illumine Nextseq 550 were analyzed by in-house IDseq software to get each microorganism’s abundance. The detail process is as follows: high-quality sequencing data were generated by removing reads of low quality or short length (<35 bp) by using fastp (Chen et al., 2018). Human host sequences were subtracted by mapping to human reference genome sequences (National Center for Biotechnology Information GRCh38 assembly) using the Burrows-Wheeler Aligner tool (BWA)^1^ (Li and Durbin, 2009). The data remaining after the removal of low-complexity reads were classified by alignment to curated microbial genome databases for viruses, bacteria, fungi, and parasites. Taxonomic references were downloaded from the National Center for Biotechnology Information.^2^

After each microorganism is quantified, it is necessary to remove the contamination from the reagent (kitome). To build the background filter for kitome removal, we categorized only the microorganisms that were detected in at least 25% of the samples, including the negative controls. Assuming that the kitome contaminants existed at a fixed abundance, the percentage of contamination would depend on and negatively correlate with the amount of extracted DNA/RNA. Pearson’s and Spearman’s correlations were used for the correlation analyses. Species that showed significant negative correlations between the abundance inferred from normalized read numbers and the extracted concentration of nucleic acids were considered reagent-derived background organisms and therefore excluded from reporting.

The conditions for selection and reporting vary for different types of microorganisms. In the case of bacteria and fungi, only outliers need to be considered, and in the case of viruses, reads need to be cover multiple areas. Microbial outliers were defined on the basis of both read-number-based and Z-score-based abundance. To minimize read-depth variation, the actual read numbers of each identified organism were normalized to reads per million total reads (RPM). The Z-score statistic was employed to identify the outliers of each organism for which abundance significantly deviated from that of the total cohort. We defined microbial outliers as those with Z-scores ≥ 2 and ≥ 10 RPM (bacteria), or ≥3 reads from distinct genomic regions (viruses), or ≥2 RPM (fungi) (Miller et al., 2019; Zinter et al., 2019a).

### Clinical Assessment

The results of PneumoSeq were reviewed along with other clinical evidence by a group of senior physicians. The clinical significance of the microorganisms detected by PneumoSeq was analyzed based on the clinical characteristics of the patient and the lung pathogenicity of the identified microorganisms (Table 2). In summary, attention was paid for common bacteria pathogens of pneumonia, such as *Streptococcus pneumoniae, Haemophilus influenzae, Moraxella catarrhalis, Klebsiella pneumoniae, Escherichia coli, Acinetobacter baumannii, pseudomonas aeruginosa, Staphylococcus aureus*, et al., if they were positive by PneumoSeq. They were considered components of the microbiological etiology of pneumonia if they were also positive in culture. When atypical pathogens, respiratory virus or pathogenic bacteria was positive by PneumoSeq, they were considered components of the etiology of pneumonia if the index patient met the clinical criteria of infection, whether confirmed by conventional tests or not. Attention was paid to most fungi other than yeast. They were considered components of the etiology of pneumonia if the index patient fulfilled risk factor and clinical criteria of pulmonary fungal disease by EORTC/MSG and responded to anti-fungal treatment, whether accompanied by microbiological or cytological evidence or not.

**TABLE 2:** Evaluation guidance for role of microorganisms detected by PneumoSeq.

**Classification of microorganisms**	**Role of microorganisms**
Common pathogens of pneumonia:	Attention was paid to these microorganisms if they were positive by PneumoSeq. They were considered components of the microbiological etiology of pneumonia if they were also positive in culture.
*Streptococcus pneumoniae, Haemophilus influenzae*, *Moraxella catarrhalis, Klebsiella pneumoniae, Escherichia coli, Acinetobacter baumannii, pseudomonas aeruginosa, Staphylococcus aureus*, et al.	
Atypical pathogens:	They were considered components of the etiology of pneumonia if the index patient met the clinical criteria of infection, whether confirmed by conventional tests or not.
*Mycoplasma pneumoniae, Chlamydia pneumoniae, Legionella pneumophila*	
Special bacteria:	They were considered components of the etiology of pneumonia if the index patient met the clinical criteria of infection, whether confirmed by conventional tests or not.
*Nocardia, Mycobacterium tuberculosis, Non-tuberculosis mycobacteria*	
Respiratory microecological bacteria	They were mostly considered normal flora
Fungus (*Candida* is excluded because primary pulmonary *Candida* pneumonia is rare)	Attention was paid to fungi. They were considered components of the etiology of pneumonia if the index patient fulfilled risk factor and clinical criteria of pulmonary fungal disease by EORTC/MSG and responded to anti-fungal treatment, whether accompanied by microbiological or cytological evidence or not.
Respiratory virus:	They were considered components of the etiology of pneumonia if the index patient met the clinical criteria of infection, whether confirmed by conventional tests or not.
Influenza A/B virus, parainfluenza-1/2/3, adenovirus, respiratory syncytial virus, human bocavirus, human metapneumovirus, human_coronavirus 229E/OC43/HKU, human rhinovirus, Cytomegalovirus, EB virus	
Microorganisms with unclear significance:	They were considered as non-pathogenic.
Torque teno virus, Torque teno mini viru, KI polyomavirus, et al.	

**FIGURE 1:**
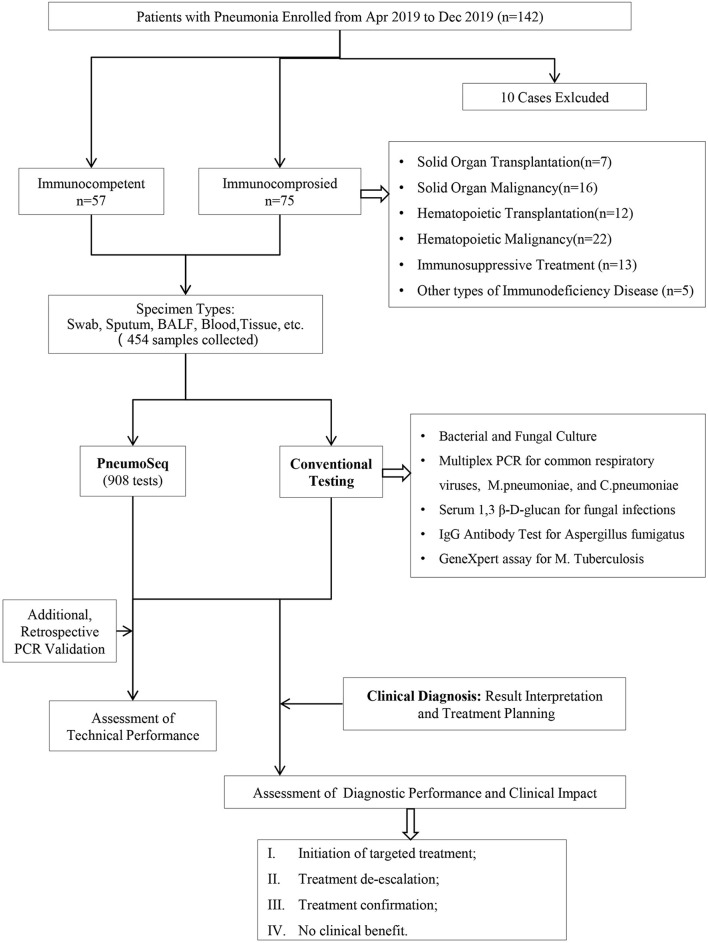
Overview of the study design. A total of 132 patients and 454 clinical specimens were tested. The performance and clinical value of PneumoSeq were evaluated based on comparisons with conventional testing and clinical diagnosis results and its impact on clinical management. BALF, bronchoalveolar lavage fluid; PCR, polymerase chain reaction.

Changes in treatment plans were made after a positive result of PneumoSeq if indicated. It should be noted that the results of throat swabs by PneumoSeq were only considered for diagnosis of respiratory viruses and/or atypical pathogens infections, but not suitable for the bacteria and fungi. After discharge, the microbiological etiology of each case and the clinical impact of PneumoSeq were assessed by the same senior physicians according to the clinical data and the guidance for the clinical benefit evaluation of PneumoSeq, which was classified as initiation of targeted treatment, pathogen identification or treatment confirmation, treatment de-escalation or no clinical benefit (Figure 1 and Table 3).

**TABLE 3:** Guidelines for evaluating the clinical benefits of PneumoSeq.

**Clinical benefit of PneumoSeq**	**Guidance from PneumoSeq in the management of patients**
Initiation of targeted treatment	PneumoSeq detected pathogens and guided targeted antimicrobial treatment.
Pathogen identification or treatment confirmation	PneumoSeq detected pathogens, either consistent with conventional tests or not, and/or PneumoSeq confirmed that the initial empirical treatment could be continued.
Treatment De-escalation	Antimicrobial drugs were de-escalated or discontinued according to the results of PneumoSeq.
No clinical benefit	Antimicrobial drugs were adjusted according to conventional tests or physician experience.

### Statistical Analysis

Comparative analyses were conducted by using the chi-square test, Fisher’s exact test for continuous variables, and Student’s *t*-test or Wilcoxon rank sum test for numerical variables where appropriate. Data analyses were performed using R software. *P* values <0.05 were considered significant, and all tests were 2-tailed unless otherwise indicated.

## Results

### Patient Characteristics and Samples

We enrolled 142 patients with pneumonia, 132 of whom completed the study (Figure 1). The median age of the 132 patients was 45 years, ranging from 14 to 83. Fifty-seven patients were immunocompetent, and seventy-five patients were immunocompromised (Figure 1 and Table 4); hematological malignancy (29%) was the most common comorbidity, followed by solid-organ malignancy (21%), immunosuppressive treatment (17%) and hematopoietic cell transplantation (16%). Twenty-eight patients were severely ill as defined by the APACHE II system, among which 6 ICHs (8%) and 2 ICOs (4%) were admitted to the ICU during hospitalization. The 30-day mortality among all study patients was 3% (Table 4). A total of 435 specimens were collected. Overall, specimens were collected from each patient, among which bronchoalveolar lavage fluid (BALF, *n* = 75), sputum (*n* = 101), plasma (*n* = 124), and nasopharyngeal swabs (*n* = 122) were the most common types. All patients were tested with PneumoSeq and conventional testing.

**TABLE 4:** Demographic data and underlying conditions of immunocompromised and immunocompetent patients with pneumonia.

**Clinical features**	**ICH**	**ICO**	***P* value**
Number of cases	75	57	NA
Sex, female	28 (37%)	28 (49%)	0.214
Age (mean ± sd)	50 ± 19	43 ± 20	0.068
Solid organ transplantation	7 (9%)	NA	NA
Hematopoietic cell transplantation	12 (16%)	NA	NA
Solid-organ malignancy	16 (21%)	NA	NA
Hematopoietic malignancy	22 (29%)	NA	NA
Immunosuppressive treatment	13 (17%)	NA	NA
Other types of immunodeficiency disease	5 (7%)	NA	NA
CTD	3 (4%)	0 (0%)	0.258
COPD	3 (4%)	5 (9%)	0.29
Hypertension	15 (20%)	7 (12%)	0.346
Diabetes	5 (7%)	4 (7%)	1
Chronic hepatic disease	8 (11%)	2 (4%)	0.186
Chronic renal disease	10 (13%)	3 (5%)	0.149
Antibiotic 30 days before onset of pneumonia	15 (20%)	0 (0%)	< 0.001
Smoker	23 (31%)	14 (25%)	0.558
Liquor	8 (11%)	3 (5%)	0.349
White blood cell counts (mean ± sd)	8.5 ± 5	9.6 ± 6.3	0.27
Neutrophil [%] (mean ± sd)	73 ± 19	86 ± 95	0.308
Lymphocyte [%] (mean ± sd)	17 ± 16	16 ± 9.5	0.781
ESR (mean ± sd)	66 ± 36	79 ± 33	0.092
C-reactive protein (mean ± sd)	10 ± 22	15 ± 28	0.471
Procalcitonin (mean ± sd)	3.2 ± 21	0.8 ± 2.5	0.342
ICU admission	6 (8%)	2 (4%)	0.465
Death within 30 days	3 (4%)	1 (2%)	0.816

*NA, the clinical features were not suitable for the control group; CTD, connective tissue diseases; COPD, chronic obstructive pulmonary disease; ESR, erythrocyte sedimentation rate; ICU, intensive care unit.*

### Development and Validation of PneumoSeq

**FIGURE 2:**
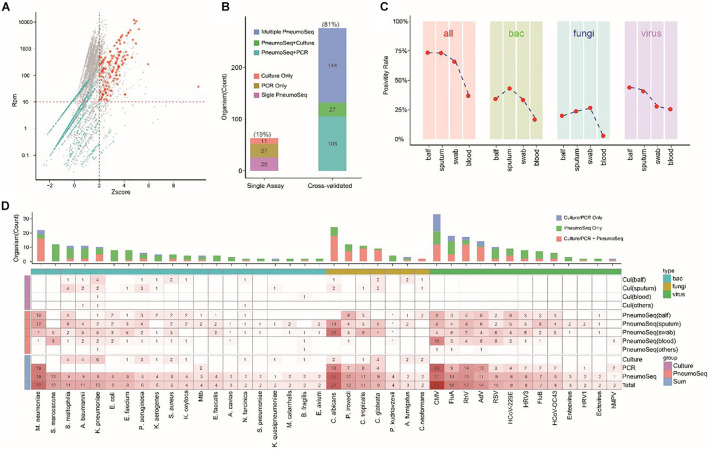
Development and validation of PneumoSeq. **(A)** Representative plot showing the identification of outlier organisms. Red, potentially pathogenic microbes that were considered outliers in terms of both abundance and read numbers. Green, all other potentially pathogenic microbes; gray, kitome contaminants that were considered background and were removed. **(B)** Cross-validation of testing results. Multiple PneumoSeq, organisms detected in multiple samples by PneumoSeq; PneumoSeq + culture and PneumoSeq + PCR, organisms detected by both PneumoSeq and culture or by both PneumoSeq and PCR, respectively. **(C)** Positivity rates of PneumoSeq results in different types of specimens. **(D)** Potential pathogens detected in our study. Bar graphs show cumulative results. Heatmap shows methods applied to various sample types. Only those microorganisms detected 2 or more times were included. *P < 0.05 by Fisher’s exact test. bac, bacteria; cul, culture; BALF, bronchoalveolar lavage fluid; PCR, polymerase chain reaction; Mtb, Mycobacterium tuberculosis; CMV, cytomegalovirus; FluA, influenza virus type A; FluB, influenza virus type B; RhV, rhinovirus; AdV, adenovirus; RSV, respiratory syncytial virus; HRV3, human respiratory virus type 3; HCoV, human coronavirus OC43; hMPV, human metapneumovirus.

The performance of the mNGS assay for diagnosing pneumonia involving various microorganisms (especially fungi with thicker cell walls) is an important metric reflecting the clinical value. Hence, we improved the assay sensitivity by establishing an effective extraction method based on a combination of bead beating, chemical lysis, and heating to ensure efficient nucleic acid extraction. As experimental reagents can cause false-positive results by introducing kitome microbes (Zinter et al., 2019b), we developed an algorithm for filtering these background species and integrated it into the PneumoSeq assay to reduce false-positive results. Furthermore, a Z-score model was employed to identify outlier microbes to achieve high assay specificity (Figure 2A).

PneumoSeq was performed on a total of 435 clinical samples. Among the 339 potential pathogens identified by PneumoSeq and/or conventional testing, 81% were cross-validated by using either another assay or other samples collected from the same patient (Figure 2B). When considering only the potential pathogens detected with PneumoSeq, we observed a cross-validation rate of 90.9% (251/276), confirming the reliability of the optimized assay.

**FIGURE 3:**
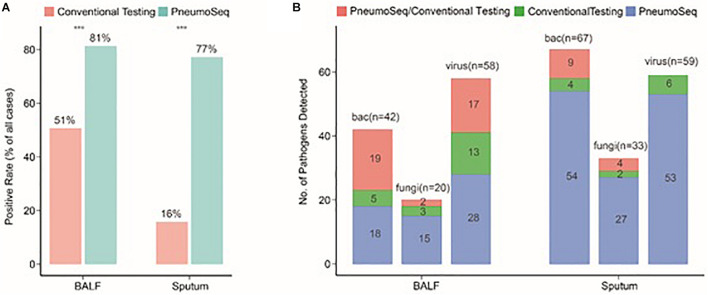
Comparison between conventional testing and PneumoSeq for pathogen detection in BALF and sputum samples. **(A)** Differences in rates of positivity by case, ***P < 0.001 by the chi-square test. **(B)** Differences in the numbers of pathogens detected. bac, bacteria; BALF, bronchoalveolar lavage fluid.

To identify the most suitable specimen type for PneumoSeq in patients with pneumonia, we compared the positivity among four major sample types. BALF and sputum were the most effective sample types for pathogen detection, showing a positivity rate of 75%, whereas only 35% of the plasma samples yielded positive results (Figure 2C). The results for nasopharyngeal swabs appeared to be comparable to those for sputum and BALF, except that a lower rate of viral detection was observed. Relative to conventional testing, the application of PneumoSeq led to significantly higher rates of pathogen detection in BALF (81 vs 51%, *P* < 0.005) and sputum samples (77 vs 16%, *P* < 2.894e-12), which was mostly attributed to the improved detection of bacterial and viral pathogens (Figure 3). As a single diagnostic assay, PneumoSeq covered a broad microbial spectrum and detected infections that were readily missed by conventional detection methods (Figure 2D).

### Microbial Spectra Differ Between ICH and ICO Patients With Pneumonia

**FIGURE 4:**
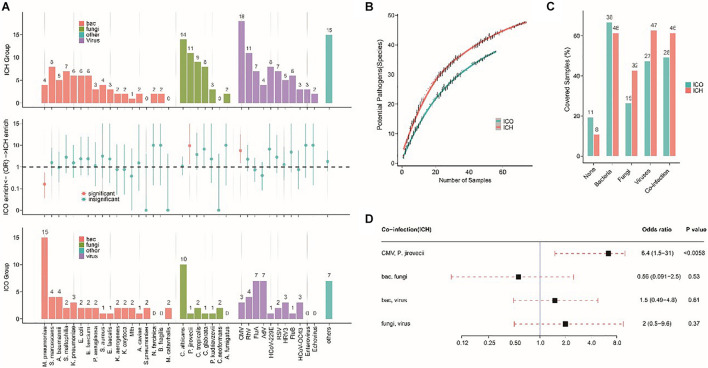
Different microbial etiologies in ICO and ICH patients with pneumonia. **(A)** Spectra of potential pathogens in ICH (upper) and ICO (lower) patients. Middle panels indicate pathogens that were significantly overrepresented (red dots, *P* < 0.05) in ICH (above the dotted line) or ICO patients (below the dotted line). **(B)** Numbers of potential pathogens identified in cumulative pneumonia cases. Random resampling of the cases at each data point was performed 50 times to generate the 95% intervals (thin lines at each point). Blue (ICO) and red (ICH) lines indicate the median value from 50 random replicates. **(C)** Distribution of different types of microbial etiology. **(D)** Potential coinfections in ICH patients. The black box indicates the median odds ratio; the red dotted line indicates the 95% confidence interval. *P*-values were calculated by Fisher’s exact tests. bac, bacteria; cul, culture; ICO, immunocompetent host; ICH, immunocompromised host; Mtb, *Mycobacterium tuberculosis*; CMV, cytomegalovirus; Flu A, influenza virus type A; Flu B, influenza virus type B; RhV, rhinovirus; AdV, adenovirus; RSV, respiratory syncytial virus; HRV3, human respiratory virus type 3; HCoV, human coronavirus OC43; hMPV, human metapneumovirus.

To further evaluate the microbial etiology of pneumonia, we compared the potential pathogens between the ICH and ICO groups. We found a differential microbial spectrum in ICH patients with pneumonia (Figure 4A). These patients exhibited significantly higher rates of *Pneumocystis jirovecii* (OR = 9.5, 95% CI, 1.3–420.7, *P* = 0.01) and CMV (OR = 5.6, 95% CI, 1.5–31.5, *P* < 0.01) infections than ICO patients, while the rate of Mycoplasma pneumoniae infections was significantly lower in ICH patients (OR = 0.16, 95% CI, 0.04–0.55, *P* < 0.001, Table 5).

**TABLE 5:** Microbial spectra differ between ICH and ICO patients with pneumonia.

**Species**	**Type**	**ICO(57)**	**ICH(75)**	**Fisher’s exact *p* value**	**Odds ratio (95% CI)**
*Mycoplasma_pneumoniae*	Bac	15	4	<0.001	0.16 (0.04–0.55)
*Serratia_marcescens*	Bac	4	8	0.552	1.58 (0.40–7.55)
*Acinetobacter_baumannii*	Bac	4	5	1	0.95 (0.19–5.01)
*Stenotrophomonas_maltophilia*	Bac	2	7	0.298	2.81 (0.51–28.80)
*Klebsiella_pneumoniae*	Bac	3	6	0.731	1.56 (0.32–10.08)
*Escherichia_coli*	Bac	2	6	0.465	2.38 (0.40–24.98)
*Enterococcus_faecium*	Bac	2	6	0.465	2.38 (0.40–24.98)
*Pseudomonas_aeruginosa*	Bac	2	3	1	1.14 (0.13–14.14)
*Staphylococcus_aureus*	Bac	1	4	0.389	3.13 (0.30–157.98)
*Enterococcus_faecalis*	Bac	1	3	0.633	2.32 (0.18–124.57)
*Klebsiella_aerogenes*	Bac	2	2	1	0.76 (0.05–10.71)
*Klebsiella_oxytoca*	Bac	2	2	1	0.76 (0.05–10.71)
*Mycobacterium_tuberculosis*	Bac	2	1	0.578	0.37 (0.01–7.36)
*Aeromonas_caviae*	Bac	1	2	1	1.53 (0.08–92.06)
*Streptococcus_pneumoniae*	Bac	2	0	0.185	0.00 (0.00–4.03)
*Nocardia_farcinica*	Bac	0	2	0.506	Inf
*Bacteroides_fragilis*	Bac	0	2	0.506	Inf
*Moraxella_catarrhalis*	Bac	2	0	0.185	0.00 (0.00–4.03)
*Candida_albicans*	Fungi	10	14	1	1.08 (0.40–2.97)
*Pneumocystis_jirovecii*	Fungi	1	11	0.013	9.51 (1.31–420.71)
*Candida_tropicalis*	Fungi	2	9	0.113	3.72 (0.73–36.75)
*Candida_glabrata*	Fungi	1	8	0.077	6.61 (0.84–301.47)
*Pichia_kudriavzevii*	Fungi	1	3	0.633	2.32 (0.18–124.57)
*Cryptococcus_neoformans*	Fungi	2	0	0.185	0.00 (0.00–4.03)
*Aspergillus_fumigatus*	Fungi	0	2	0.506	Inf
CMV	Virus	3	18	0.004	5.62 (1.52–31.46)
Rhinovirus	Virus	4	11	0.268	2.26 (0.62–10.32)
Influenza_A_virus	Virus	7	7	0.584	0.74 (0.21–2.64)
Human_adenovirus	Virus	7	4	0.206	0.41 (0.08–1.69)
Human_coronavirus_229E	Virus	1	8	0.077	6.61 (0.84–301.47)
Human_respiratory_syncytial_virus	Virus	2	7	0.298	2.81 (0.51–28.80)
Human_respirovirus_3	Virus	3	5	1	1.28 (0.24–8.62)
Influenza_B_virus	Virus	1	6	0.140	4.82 (0.56–227.67)
Human_coronavirus_OC43	Virus	3	3	1	0.75 (0.10–5.83)
Enterovirus	Virus	0	3	0.258	Inf
Echovirus	Virus	0	2	0.506	Inf
Other	Other	7	15	0.346	1.78 (0.62–5.58)

*ICH, immunocompromised host; ICO, immunocompetent host; CMV, cytomegalovirus.*

**FIGURE 5:**
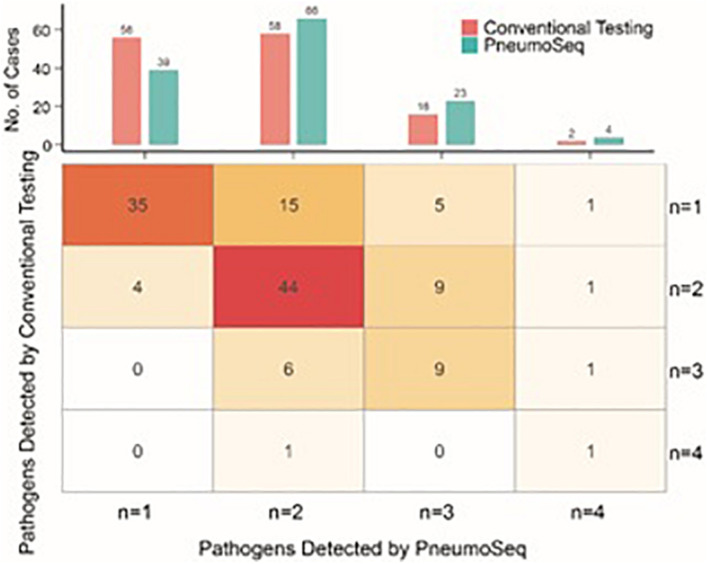
Comparison between conventional testing and PneumoSeq for detecting potential polymicrobial infections. Upper panel, cases with various numbers of potential pathogens detected; Lower panel, expanded table showing the detailed case distribution.

The simultaneous detection of two or more pathogens was more common in ICH patients with pneumonia, with a significantly greater number of potential pathogens being identified in each patient than in their ICO counterparts (2.7 vs 1.8, *P* < 0.01, Figure 5). The overall diversity of pathogens was also greater in ICH patients with pneumonia (Figure 4B, *P* < 0.01). When grouped by microbial type, ICH patients with pneumonia showed a higher percentage of viral, fungal, and polymicrobial infections but a slightly lower percentage of bacterial infections (Figure 4C). Moreover, *P. jirovecii* infections were more significantly overrepresented in ICH patients with CMV (OR = 6.4, 95% CI, 1.5–31.0, *P* < 0.01, Figure 4D).

### Assessment of the Real-World Clinical Impact of PneumoSeq

**FIGURE 6:**
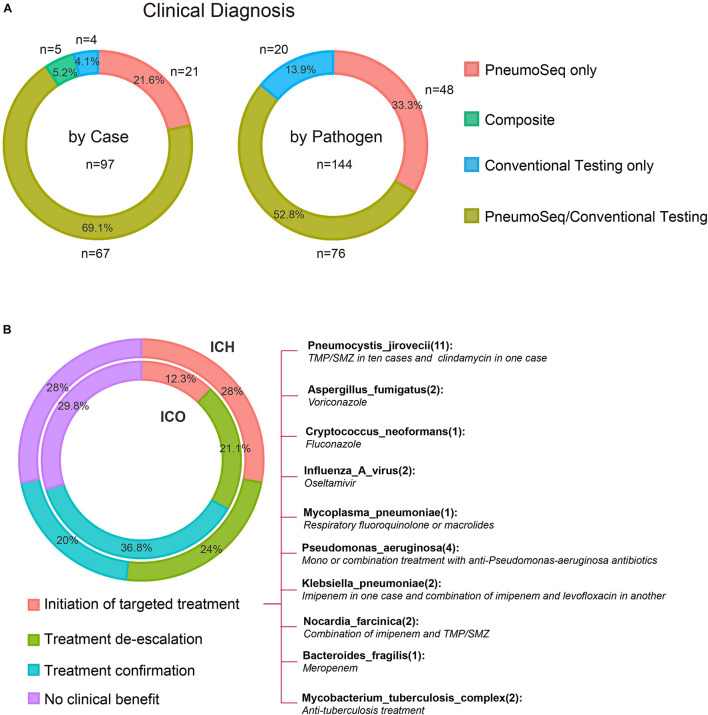
Diagnostic performance and the real-world clinical impact of PneumoSeq. **(A)** Agreement between clinical diagnosis and testing results. Analyses were performed in 97 patients whose microbial etiology was diagnosed clinically, involving a total of 144 pathogens. PneumoSeq/Conventional Testing, pathogens identified by both methods in agreement with clinical diagnosis; Composite, pathogens identified by combining PneumoSeq and the conventional testing results; PneumoSeq Only and Conventional Testing Only, pathogens identified by an only single method. Left panel, by case; Right panel, by microorganism. **(B)** Evaluation of the clinical impact of PneumoSeq in ICO and ICH patients. Inner circle, ICO; Outer circle, ICH. The clinical impact was assessed as 1 of the 4 categories indicated with different colors.

**FIGURE 7:**
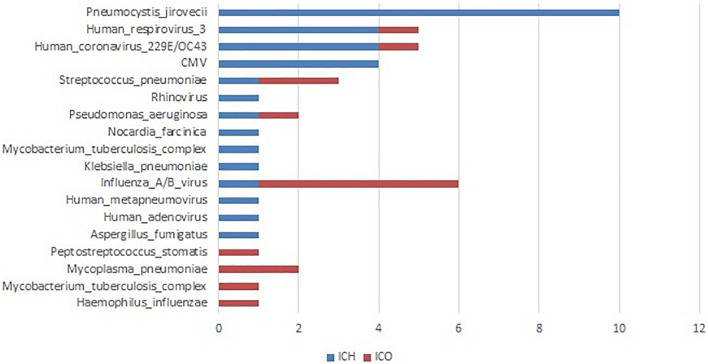
Clinically determined pathogens only detected by PneumoSeq, not by conventional methods in pneumonia patients. ICH, immunocompromised host; ICO, immunocompetent host; CMV, cytomegalovirus.

All patients underwent both conventional and PneumoSeq testing. Among the 97 patients who received clinical diagnoses of the causative pathogens (73.5%, 97/132), 90.7% (88/97) of the diagnoses were supported by PneumoSeq. Conventional testing yielded a missed diagnosis in 16.7% of the cases (22/132), whereas conventional testing detected pathogens in 2.3% (3/132) of the cases that were deemed negative by PneumoSeq. Similarly, among all clinically diagnosed pathogens, 86.1% (124/144) showed findings consistent with those of PneumoSeq. Importantly, PneumoSeq reported an additional 48 pathogenic microorganisms that could have been missed if using only conventional methods (Figure 6A and Figure 7).

We finally attempted to assess the clinical impact of the assay’s findings. PneumoSeq led to an overall beneficial impact on treatment in 33.3% (19/57) of the ICO patients and 52.0% (39/75) of the ICH patients. The beneficial impacts were categorized as either the successful de-escalation of treatment (*n* = 12 in ICO, *n* = 18 in ICH) or the initiation of targeted treatment (*n* = 7 in ICO, *n* = 21 in ICH). PneumoSeq also helped identify the etiology or confirm appropriate treatment in 36.8% (21/57) and 20.0% (15/75) of the ICO and ICH patients, respectively. PneumoSeq showed no clinical benefit because of failure in identifying pathogens or guiding targeted antimicrobial treatment in slightly less than 30% of both ICO and ICH patients (Figure 6).

## Discussion

By enrolling both ICO and ICH patients with pneumonia and testing for multiple types of specimens in the same individual patient, we evaluated the value of PneumoSeq for the diagnosis of pneumonia and assessed its clinical impact in a real-world setting in both ICO and ICH patients. We found that PneumoSeq detected additional pathogens in 22 patients, including CMV, *P. jirovecii*, *Nocardia*, and fungi in the ICH group and influenza virus, *M. pneumoniae*, and *S. pneumoniae* in the ICO group, lead to de-escalation of antimicrobial treatment and initiation of appropriate treatment.

The rate of confirmed etiologies in our study was higher than that previously reported (77 vs 44% in ICH, 77 vs 32% in ICO) (Sousa et al., 2013), with a considerable proportion of polymicrobial infections (40% in ICH and 15.8% in ICO). PneumoSeq identified potential pathogens in 70.5% of the cases. Missed infections were suggested in 22 patients with negative results according to all other conventional assays. The additional pathogens detected by PneumoSeq included CMV, *P. jirovecii*, *Nocardia*, and fungi in the ICH group and influenza virus, *M. pneumoniae*, and *S. pneumoniae* in the ICO group. The reasons for false negative of these pathogens by traditional diagnostic methods included lack of detection technology, such as authenticated pneumocystis nucleic acid detection assay, low positivity in pathology or cytology for fungi, *P. jirovecii*, etc., low positivity in culture for *Nocardia*, *P. streptococcus*, etc. Moreover, some pathogens such as CMV, *M pneumoniae*, etc. was not considered by clinicians during hospital addition based on empirical judgments, and therefore conventional assays were not performed.

For the above-mentioned pathogens additionally detected by PneumoSeq, targeted drugs are available for clinician to make personalized treatment for patients. Hence, the improved ability to identify the microbial etiology by using PneumoSeq further enabled the application of targeted treatment in 12.3% of ICO patients and 28% of ICH patients. These results suggest the value of PneumoSeq in guiding the clinical management of pneumonia, especially in ICH patients. The clinical benefit of mNGS observed in our study was superior to its reported utility in a prospective study for diagnosing meningitis (14.3%) (Wilson et al., 2019) and that of plasma cell-free DNA in a retrospective study for exploring the pathogens of infectious diseases (7.3%) (Hogan et al., 2020). There was a lack of prospective studies in the literature on the application of mNGS in lung infectious diseases. The existing data were basically retrospective studies, exploring the value in pathogenic diagnosis. There were limited data to assess whether the patients will benefit from the result of mNGS (Chen et al., 2021; Peng et al., 2021; Zhang et al., 2021).

Our data also showed a different spectrum of pathogens in ICH patients with pneumonia than in their ICO counterparts. ICH patients had a more complex microbial etiology, with higher detection rates of fungal, viral and coexisting infectious agents, such as CMV and *P. jirovecii*. These findings emphasized the challenges in the diagnosis of ICHs with pneumonia by conventional testing. PneumoSeq improved yield of pathogens and lead to personalized targeted antimicrobial treatment in ICH patients.

Kalantar et al. (2019) applied mNGS to compare the yield of tracheal aspirate and minibronchoalveolar lavage (mBAL) specimens in the assessment of the respiratory microbiota of ventilated patients, and they found that the use of tracheal aspirates provided a similar assessment of airway microbiota to that obtained using mBAL in patients with pneumonia (Kalantar et al., 2019). By comparing the PneumoSeq results from tests of different specimen types, BALF and sputum samples were found to be the most appropriate sample types for pathogen detection, with BALF showing less contamination by oral microorganisms. In patients whose BALF is unavailable at a particular time, sputum can serve as a promising alternative. Although nasopharyngeal swabs show a slightly lower positivity rate, they could serve as a practical alternative sample type when neither of the other two types is available.

Although PneumoSeq showed a higher diagnostic performance than other conventional methods, as a high-throughput sequencing-based assay, its sensitivity could be affected by the abundance of pathogens and the host cell content in each sample. While PneumoSeq identified more pathogens than other conventional methods combined, potential false-negative results were also observed, as suggested by other assays. This could be due to the high abundance of host cells in these samples (Chiu and Miller, 2019; Miller et al., 2019), which limited the detection ability of PneumoSeq to a greater degree than that of other targeted methods, such as PCR. This should be noted when interpreting results from any metagenomic-based assay.

As a promising diagnostic tool, the wider application of mNGS is still hindered by its cost and turnaround time (Flygare et al., 2016). Currently, an mNGS assay costs $1000–2000 in the US and $400–500 in China (Miao et al., 2018; Ramachandran and Wilson, 2020), and the turnaround time is usually 24–48 h (Simner et al., 2018). However, it is cheaper than the total conventional microbiological testing cost of approximately $1000. As the network of clinical laboratories that offer this new assay expands, its affordability and feasibility are expected to be improved in clinical settings (Simner et al., 2018; Han et al., 2019).

mNGS is unique in that it collects unbiased sequence information of pathogens as well as host genes (Avraham et al., 2016; Chiu and Miller, 2019). Some previous studies have shown that this information could be used for analyses of drug resistance and virulence genes and even host responses and that it offers added diagnostic value (Fournier et al., 2014; Forbes et al., 2017; Chiu and Miller, 2019). More studies are still needed for the validation of these assays and the assessment of their clinical impact.

Our study has limitations as a single-center study in which all patients were enrolled from a single institution. Some patients had received ineffective treatment prior to being referred to our hospital. This might have resulted in a decreased positivity rate by culture and a higher chance of infections caused by opportunistic pathogens.

In conclusion, our study has demonstrated the clinical value of PneumoSeq for pathogen detection and management in ICH and ICO patients with pneumonia.

## Data Availability Statement

The data can be found in the China National GeneBank DataBase (CNGBdb), which is a unified platform for the scientific research community to provide biological big data sharing and application services (https://db.cngb.org/search/project/CNP0002153/).

## Ethics Statement

The studies involving human participants were reviewed and approved by Ethics Committee of the First Affiliated Hospital of Guangzhou Medical University. Written informed consent to participate in this study was provided by the participants’ legal guardian/next of kin.

## Author Contributions

YZ, TX, FH, and MX contributed to conception and design of the study. ZL and SL organized the database. FH, XL, and MX performed the statistical analysis. YZ and FH wrote the first draft of the manuscript. TX and W-JG wrote sections of the manuscript. All authors contributed to manuscript revision, read, and approved the submitted version.

## Conflict of Interest

TX, FH, MX, and XL are employed by Vision Medicals Co., Ltd., Guangzhou, Guangdong Province, China. The remaining authors declare that the research was conducted in the absence of any commercial or financial relationships that could be construed as a potential conflict of interest.

## Publisher’s Note

All claims expressed in this article are solely those of the authors and do not necessarily represent those of their affiliated organizations, or those of the publisher, the editors and the reviewers. Any product that may be evaluated in this article, or claim that may be made by its manufacturer, is not guaranteed or endorsed by the publisher.

## Footnotes

1
http://bio-bwa.sourceforge.net/

2
ftp://ftp.ncbi.nlm.nih.gov/genomes/
